# Individual-level quality-of-life changes are common after aneurysm screening in women: A prospective cohort study

**DOI:** 10.1016/j.bas.2026.107491

**Published:** 2026-10-23

**Authors:** Andrei Schildt, Ville Vasankari, Jussi Numminen, Mika Niemelä, Miikka Korja, Justiina Huhtakangas

**Affiliations:** Department of Neurosurgery, Helsinki University Hospital & University of Helsinki, Helsinki, Finland

**Keywords:** Intracranial aneurysm, Mass screening, Quality of life, Anxiety, Cohort studies, Smokers

## Abstract

**Introduction:**

Screening for intracranial aneurysms enables the treatment of lesions at risk of rupture, but concerns remain about its psychological effects. We investigated the impact of screening on health-related quality of life (HRQoL) in 50–60-year-old female smokers.

**Research question:**

How does aneurysm screening affect HRQoL?

**Methods:**

HRQoL was prospectively assessed using 15D and EQ-5D-3L before, 3 months, and 12 months after screening. Changes in individual HRQoL domains were analyzed, and MIC thresholds were used to define clinically relevant changes in the EQ index and EQ visual analogue scale (EQ VAS). Linear mixed models determined whether the timepoint, computed tomography angiography (CTA) results, or their interaction affected EQ outcomes.

**Results:**

Of 108 women, 13 had abnormal vascular findings, and two underwent surgery. Three-month follow-up questionnaires were returned by 92 participants and 57 completed the 12-month follow-up. Linear mixed models showed no effect of timepoint, CTA results, or their interaction on EQ index or EQ VAS. Nevertheless, 54 participants (59%) deteriorated in at least one 15D domain, including depression (n = 14), sleep (n = 12), and anxiety (n = 9). Sixty-seven reported improvements, notably in sleep (n = 22), symptoms and discomfort (n = 19), and vitality (n = 19). By MIC thresholds, 11 participants showed clinically significant EQ index worsening and nine improvement.

**Discussion and conclusion:**

Overall EQ-5D scores suggested no HRQoL impairment, but individual-level analyses revealed worsening of anxiety and depression irrespective of screening results. Standard HRQoL instruments may miss psychosocial effects of screening. Future studies should incorporate pre-invitation baseline assessments and anxiety-specific measures alongside generic HRQoL questionnaires.

## Highlights

• Screening of 108 female smokers for UIAs caused no significant HRQoL change.
• Thirteen participants had abnormal vascular findings, two underwent surgery.
• Over half of participants worsened in some 15D domain, including mental health.
• Targeted anxiety tools (GAD-7, HADS) may better capture screening effects.

## 1 Introduction

Aneurysmal subarachnoid haemorrhage (aSAH) carries exceedingly high mortality and long-term disability rates (Asikainen et al., 2023; Wang et al., 2026; Suarez et al., 2006; Roquer et al., 2020). As aneurysms are often asymptomatic and therefore rarely detected prior to rupture, preventative screening programs of individuals with a high prevalence of unruptured intracranial aneurysms (UIAs) using noninvasive imaging methods have been recommended (Ogilvy, 2025; Thompson et al., 2015). Currently, the American Heart Association (AHA) recommends screening of people with familial history, autosomal dominant polycystic kidney disease (ADPKD) and other pathologies associated with an increased UIA prevalence (Thompson et al., 2015).

Although screening enables the pre-emptive treatment of aneurysms deemed likely to rupture, it may expose patients to unnecessary harm resulting from overtreatment, since a minority of all UIAs rupture in lifetime (Korja et al., 2014). Furthermore, knowledge of harboring an UIA may cause significant psychological distress. This may be reflected in health-related quality of life (HRQoL), as HRQoL measures such as the EQ-5D and 15D have been found to be affected by clinically diagnosed anxiety and depression (Saarni et al., 2007; Liao et al., 2021). Previous studies have also found that patients with incidentally discovered UIAs have both a decreased quality of life and/or psychiatric issues compared to the general population (Lemos et al., 2020; Jelen et al., 2023; Algra et al., 2022; Yoshimoto and Tanaka, 2013; Wenz et al., 2016; Buijs et al., 2012; Li et al., 2017).

However, evidence on aneurysms detected through screening is less consistent. In a Norwegian nested case-control study, a UIA diagnosis was generally not associated with worse HRQoL several years after magnetic resonance angiography (MRA) screening (Rosenlund et al., 2024). Similarly, screening of relatives of patients with UIAs had no negative effect on HRQoL over one year (Mensing et al., 2023). Data on changes in quality of life during the short-term period surrounding targeted screening of high-risk groups remain scarce. Other factors may also be tied to the HRQoL of UIA-screened cohorts. For example, smoking itself is consistently associated with decreased HRQoL (Zhong et al., 2025; Nolazco et al., 2024; Rezaei et al., 2017).

Utilizing prospectively collected data from a previously completed screening study of 50- to 60-year-old female smokers (Huhtakangas et al., 2025), we aim to quantify the effect of UIA screening on HRQoL. Especially, we seek to compare the HRQoL impact on participants with positive and negative screening findings. We hypothesize that screening will result in a significant decrease in HRQoL for participants with abnormal vascular computed tomography angiography findings due to an increase in psychological distress. Likewise, we hypothesize that the HRQoL of those with normal CTA findings is likely to remain relatively stable at 1-3 months following screening.

## 2 Methods

### 2.1 Study design and population

The full study protocol has been described previously (Huhtakangas et al., 2021). Shortly, smokers were identified through either the Finnish Institute for Health and Welfare (THL) biobank, or through the Helsinki Biobank. These participants were then sent preliminary invitations. Those who expressed preliminary interest in joining the study were then sent full descriptions of the study protocol, 15D and EQ-5D-3L questionnaires, instructions, consent and contact details. Participants with kidney failure and iodinated contrast allergy were excluded.

Participants were recruited between 2019 and 2024. Participants reported their HRQoL in the form of the 15D and EQ-5D-3L questionnaires. The questionnaires were sent by mail to each participant following initial consent, with instructions to self-book the CTA. Following imaging, patients were informed of the outcome and sent the same HRQoL questionnaires. Questionnaires were returned within three months. Participants recruited in 2019-2020 (n = 43) were sent a follow-up questionnaire at five years, and those recruited in 2023-2024 (n = 65) at 12 months. This observational study is reported in accordance with Strengthening the Reporting of Observational Studies in Epidemiology (STROBE) Statement (Vandenbroucke et al., 2007), and the full checklist may be found in Supplementary File 1.

All participants gave their informed consent; ethical approval was obtained from the Helsinki University Hospital Ethics Committee (HUS/1144/2022).

### 2.2 Health-related quality of life questionnaires

HRQoL was evaluated using two separate instruments: the 15D (Sintonen, 2001) and EQ-5D-3L (Brooks, 1996) questionnaires. The 15D instrument was developed in Finland and includes 15 different dimensions of quality of life: mobility, vision, hearing, breathing, sleeping, eating, speech, excretion, usual activity, mental function, discomfort and symptoms, depression, anxiety, vitality and sexual activity (Sintonen, 2001). Respondents rate their current status on a five-level ordinal scale for each dimension, with one representing no disability in the selected domain and five indicating severe disability. These responses are then combined into a single 15D index score ranging from 0 to 1.

In contrast, the EQ-5D-3L instrument consists of five dimensions: mobility, self-care, anxiety/depression, usual activities and pain/discomfort (Brooks, 1996). Participants rate each dimension based on a three-level ordinal scale, with one representing no problems, two representing some problems and three indicating severe problems in the selected domain. These ratings are converted into a single index score (0 to 1) using country-specific value sets (EuroQol Research Foundation, 2026). For the purposes of this study, a value set for Finland was used (Ohinmaa and Sintonen, 1999). Additionally, as part of the EQ-5D-3L, participants self-report their overall health status on a 0 to 100 visual analogue scale (EQ VAS). The EQ VAS entails participants rating their own health on a linear scale, with one endpoint representing the best imaginable state of the health and the other endpoint indicating the worst possible health state.

To identify changes in the EQ index or EQ VAS significant enough to cause a perceivable change in the responder's wellbeing, a Minimal Important Change (MIC) cutoff value was used. As no estimates specific to middle-aged female smokers undergoing aneurysm screening exist, we used a threshold of 0.074 for the EQ index, the mean MID reported by Walters and Brazier across 11 patient groups from eight longitudinal studies (range −0.011 to 0.140) (Walters and Brazier, 2005). This means that a positive or negative change in the EQ index (ranging 0 to 1) exceeding this value is considered a clinically significant change. For the EQ VAS we used the pooled estimates of Cheng et al., who reported a mean MID of 9.2 for improvement and −6.5 for deterioration (Cheng et al., 2024).

### 2.3 Statistical analysis

Continuous variables were checked for normality using the Shapiro-Wilk test for normality. To test for differences in baseline characteristics between participants with normal and abnormal CTA results, independent samples T-test was used for normally distributed variables and Mann-Whitney *U* test was used for non-normal variables. Baseline characteristics are reported in medians and interquartile ranges (IQR). Categorical variables are reported as frequencies and percentages and were compared using Pearson's Chi-square or Fisher's exact test.

To evaluate changes in HRQoL over time and whether these differed according to CTA findings, Linear Mixed Models (LMM) were utilized for both the EQ index and EQ VAS. The model was designed to evaluate the independent impact of the screening result (normal vs. abnormal) and the specific timepoint (baseline vs. three-month follow-up). Furthermore, a group-by-time interaction term was included to test specifically whether the trajectory of HRQoL changes between the two timepoints differed depending on the patient's CTA result. Patient age at the time of the CTA was included as a covariate to adjust for baseline age-related variance.

Because aggregate HRQoL indices may dilute significant variations in specific, screening-relevant psychological domains (such as anxiety and depression), and acknowledging the limited statistical power to detect definitive group differences with a small cohort of abnormal CTA findings, participant-level descriptive analyses were prioritized.

Changes across individual 15D and EQ-5D-3L domains were calculated to identify the specific proportion of participants experiencing deterioration or improvement. Missing data were handled by available-case analysis. A participant contributed to a given comparison if both the baseline and the follow-up value for that specific item were recorded; no imputation was performed. MIC thresholds were applied to identify how many patients in each respective group experienced clinically meaningful shifts in their HRQoL following screening.

Statistical significance was defined as a two-tailed p-value < 0.05. All analyses were performed using IBM SPSS Statistics, version 31.0.2.0 (IBM Corp., Armonk, N.Y., USA).

### 2.4 Sources of bias

To address potential attrition bias, baseline characteristics and HRQoL were compared between responders and non-responders at both the three-month follow-up and the 12-month follow-up using the tests described in section 2.3. Linear mixed models were used because they include all available observations and give unbiased estimates when data are missing at random. To reduce the influence of random fluctuation, a clinically important change was defined using published MIC thresholds rather than any change in score. As there was no unscreened control group, individual-level results were analyzed descriptively and not interpreted causally.

## 3 Results

Of the 458 preliminary invitations sent, 160 women replied, 116 returned the questionnaires and signed consent, while a total of 108 participants ultimately underwent CTA screening. Following screening, 92 participants (85%) returned the three-month questionnaire. A 12-month follow-up was collected for a portion of the cohort (n = 57, 53%), whereas a 5-year follow-up was collected for another portion. Long-term (5-year) follow-up results were excluded, as factors affecting quality of life during the 5-year long follow-up period are highly unlikely to be related to the screening itself. The full HRQoL data collection process is illustrated in Fig. 1.

**Fig. 1:**
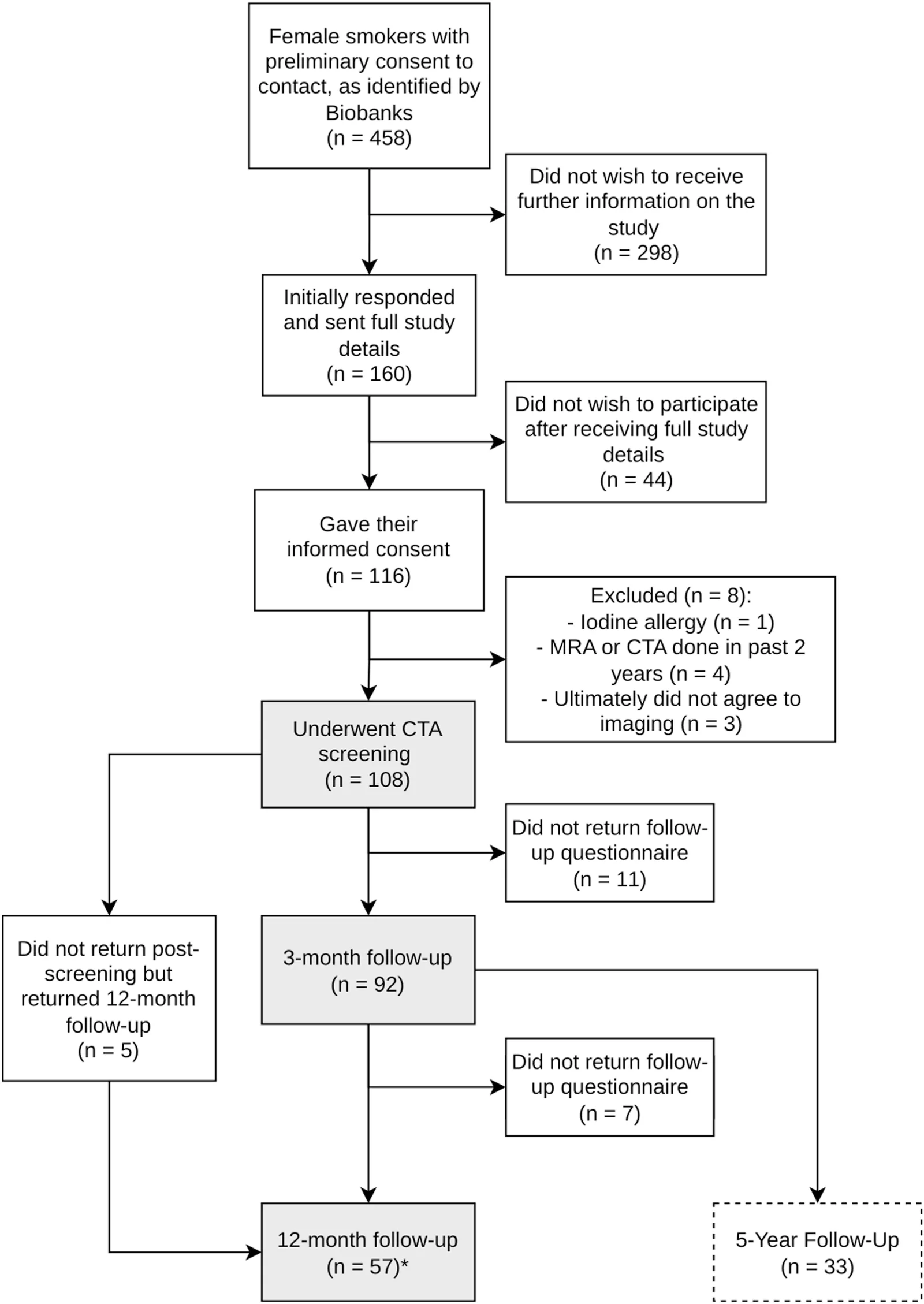
Response rate to HRQoL questionnaires across study phases. *HRQoL data available at all timepoints (baseline, 3- and 12-month follow-up) for 52 participants (48%). MRA = magnetic resonance angiography; CTA = computed tomography angiography.

Differences between responders and non-responders were compared for both 3- and 12-month follow-ups. Three-month non-responders were younger than responders (median 53.3 vs 57.1 years, p = 0.003) but did not differ in other baseline characteristics, baseline HRQoL or CTA result (Supplementary Table S1A). The 12-month questionnaire was sent to the 65 participants of the 2023–2024 wave, of whom 57 (88%) returned it; 52 of these also had three-month data. In this wave, responders and non-responders did not differ in baseline characteristics or three-month HRQoL (Supplementary Table S1B).

### 3.1 Baseline characteristics

Thirteen out of 108 screened participants had abnormal vascular imaging findings, of which one was a dural arteriovenous fistula (dAVF) and the rest UIAs, of which one was extradural. Two participants were found to have meningiomas. One of these meningiomas was previously diagnosed, and both were small and asymptomatic. Therefore, these patients were categorized as having normal CTA results for the purposes of our analysis.

The median age at CTA was 56.4 (IQR 5.8), and the median smoking duration was 37 years (IQR 10). Thirteen (12%) participants reported having previously diagnosed depression, all of which had normal CTA findings. At baseline, no statistically significant differences were found in age (p = 0.236), BMI (p = 0.466), hypertension (p = 0.822), hypercholesterolemia (p = 0.369), asthma and/or COPD (p = 0.480), depression (p = 0.359), cancer (p = 0.345), smoking duration (p = 0.593), cigarettes per day (p = 0.730), pack-years (p = 0.899) or baseline HRQoL (EQ index p = 0.119, EQ VAS p = 0.281) between those with normal and non-normal CTA results. The baseline characteristics are presented for the total cohort and by group in Table 1.

**Table 1:** Baseline characteristics of the participants.

	Total cohort (n = 108)	Normal CTA (n = 95)	Abnormal CTA (n = 13)	P-value
**Demographics**				
Age at CTA, y (IQR)	56.4 (5.8)	56.0 (5.8)	58.0 (3.7)	0.236
BMI, kg/m^2^* (IQR)	27.1 (8.6)	27.0 (8.5)	28.1 (10.2)	0.466
**Comorbidities**				
Hypertension	55 (50.9%)	48 (50.5%)	7 (53.8%)	0.822
Hypercholesterolemia	39 (36.1%)	36 (37.9%)	3 (23.1%)	0.369
Asthma and/or COPD	24 (22.2%)	20 (21.1%)	4 (30.8%)	0.480
Depression	13 (12.0%)	13 (13.7%)	0 (0.0%)	0.359
Cancer	10 (9.3%)	8 (8.4%)	2 (15.4%)	0.345
**Baseline smoking history**				
Smoking duration*, y (IQR)	37.0 (10.0)	36.5 (10.0)	40.0 (9.0)	0.593
Cigarettes per day^‡^, n (IQR)	13.0 (5.0)	15.0 (7.0)	12.3 (5.0)	0.730
Pack-years^†^ (IQR)	22.1 (18.0)	22.3 (18.9)	20.0 (8.1)	0.899
**Baseline HRQoL**				
EQ VAS score^‡^ (0-100)	80.0 (15.0)	80.0 (20.0)	85.0 (20.0)	0.281
EQ index* (0-1)	0.73 (0.26)	0.68 (0.11)	0.73 (0.33)	0.119

Abbreviations: CTA = computed tomography angiography; BMI = body mass index; HRQoL = health-related quality of life; COPD = chronic obstructive pulmonary disease *Data were missing for 3 participants † Data were missing for 4 participants ^‡^Data were missing for 1 participant.

### 3.2 Participant-level changes in 15D dimensions

#### 3.2.1 Changes at 3 months

At three months following screening, 54 (59%) out of 92 participants experienced worsening in one or more 15D domains when compared to baseline (49 with normal CTA, 5 with abnormal vascular findings). A greater number of participants (n = 67; 63 normal, 4 abnormal) experienced improvement in at least one domain. Many participants experienced worsening in mental health-related domains, such as depression, sleep and anxiety. However, improvements were also prominent in these same domains, and significant variation was seen in other seemingly unrelated domains. Changes in HRQoL domains occurred in broadly similar proportions regardless of CTA finding. Participant-level counts for the 15D domains at both timepoints are summarized in Table 2.

**Table 2:** Participant-level changes across all 15D domains. Values are the number of participants reporting a change (normal CTA/abnormal CTA). Three-month changes are relative to baseline (n = 92); 12-month changes are relative to the three-month assessment. Both twelve- and three-month data were available for a subset of the cohort (n = 52).

15D domain	Worsened at 3 mo	Improved at 3 mo	Worsened at 12 mo	Improved at 12 mo
Mobility	4 (3/1)	8 (8/0)	6 (5/1)	3 (3/0)
Vision	10 (8/2)	8 (8/0)	7 (5/2)	11 (10/1)
Hearing	7 (7/0)	5 (5/0)	2 (2/0)	2 (2/0)
Breathing	10 (9/1)	13 (13/0)	5 (5/0)	8 (8/0)
Sleeping	12 (11/1)	22 (21/1)	16 (15/1)	11 (11/0)
Eating	1 (1/0)	2 (2/0)	1 (1/0)	1 (1/0)
Speech	1 (1/0)	3 (3/0)	2 (2/0)	1 (1/0)
Excretion	8 (6/2)	12 (12/0)	9 (8/1)	7 (6/1)
Usual activities	9 (8/1)	8 (6/2)	10 (9/1)	8 (8/0)
Mental function	8 (6/2)	9 (9/0)	7 (7/0)	9 (8/1)
Discomfort and symptoms	10 (9/1)	19 (17/2)	12 (10/2)	8 (8/0)
Depression	14 (12/2)	12 (11/1)	13 (12/1)	7 (7/0)
Anxiety	9 (7/2)	11 (11/0)	12 (11/1)	7 (5/2)
Vitality	9 (7/2)	19 (18/1)	11 (10/1)	7 (7/0)
Sexual activity	7 (6/1)	8 (8/0)	7 (7/0)	5 (5/0)

The average 15D dimensions for patients with normal and abnormal CTA findings are visualized in Fig. 2., and the number of participants experiencing changes in each corresponding 15D dimension are shown in Fig. 3.

**Fig. 2:**
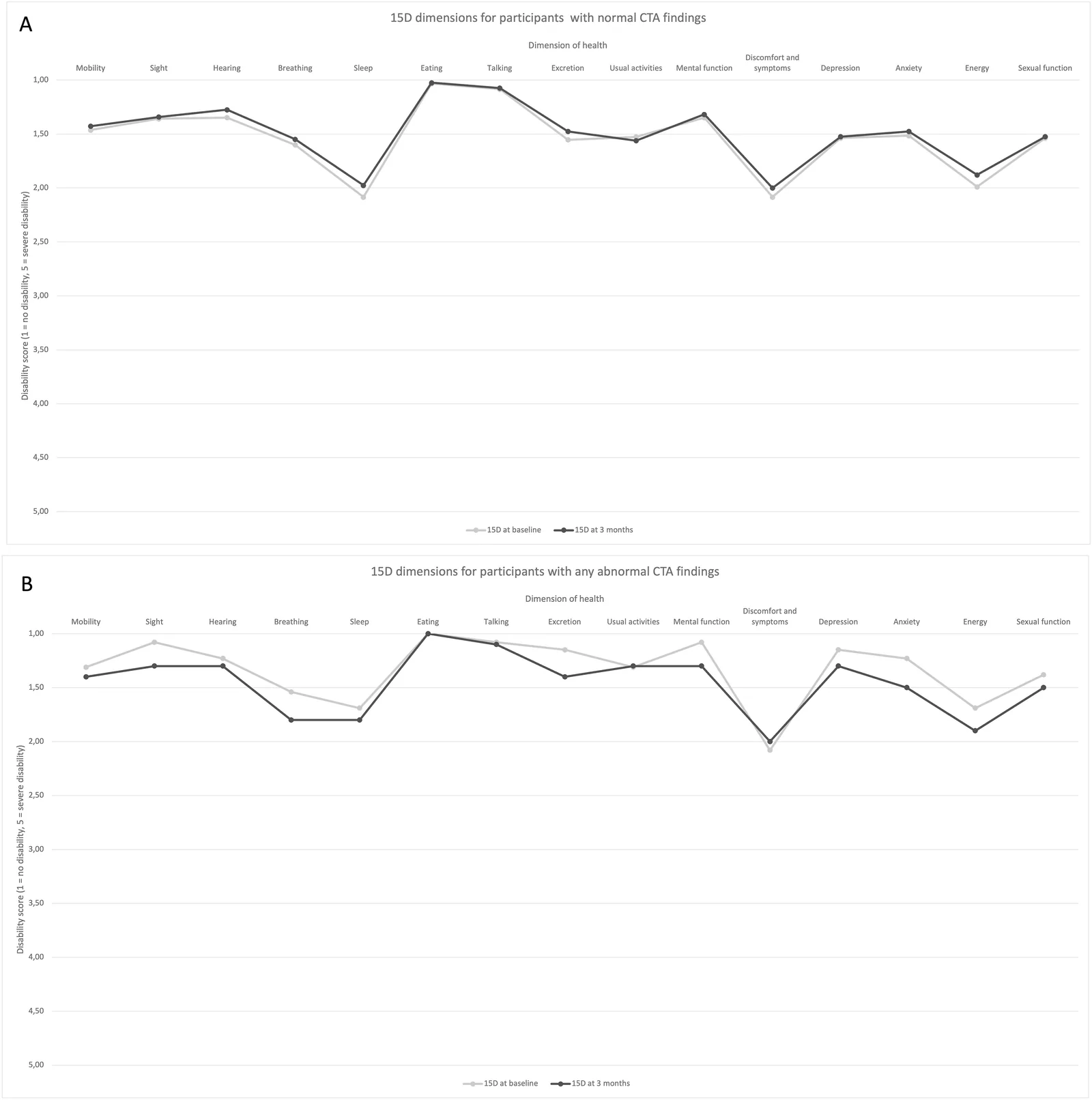
Mean HRQoL results as per the 15D dimensions at baseline and at three months following screening for A) participants with normal CTA findings and B) participants with any abnormal CTA findings. A score of 1 indicates no disability in the selected domain while a score of 5 indicates severe disability.

**Fig. 3:**
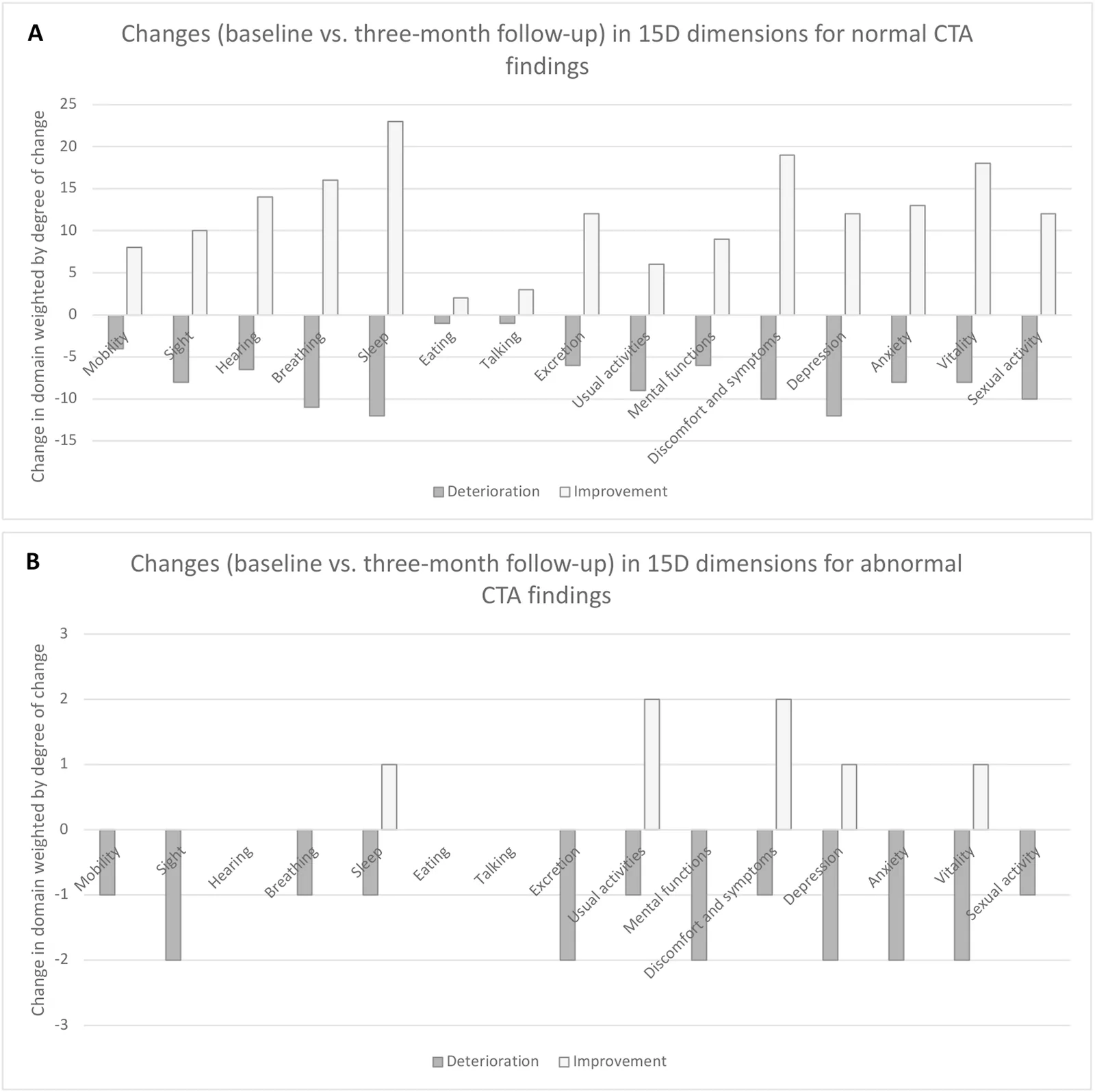
Participant-level changes in each corresponding 15D domain. Worsening in any domain is expressed as a negative value, improvements positive. Changes are weighted by the degree of change in that domain (1 to 5).

#### 3.2.2 Changes at 12 months

Comparing the three- and 12-month scores in those participants for whom the 12-month follow-up was available, 39 (75%) out of 52 participants reported worsening (35 normal, 4 abnormal) and 41 (79%) reported improvements in at least one 15D domain. Like at the three-month follow-up, worsening was notable in sleep, depression and anxiety, whereas improvement in depression and sleep was confined to participants with normal CTA findings. Full per-domain counts are presented in Table 2.

### 3.3 Participant-level changes in EQ-5D-3L and EQ VAS

#### 3.3.1 Changes at 3 months

At three months, 15 out of 91 participants (17%; 14 normal, 1 abnormal) reported worsening and 19 (21%; all normal) reported improvements in one or more EQ-5D-3L domains relative to baseline. Within the psychological domain, anxiety and depression worsened in 8 participants (7 normal, 1 abnormal) and improved in 5 (all with normal CTA findings). Applying the MIC thresholds, 11 participants crossed the threshold for a clinically significant worsening of the EQ index (10 normal, 1 abnormal) and 9 for improvement (all normal); for the EQ VAS, 14 worsened and 25 improved. Participant-level counts for the EQ-5D-3L domains, together with the numbers of participants crossing the MIC thresholds, are summarized in Table 3.

**Table 3:** Participant-level changes across EQ-5D-3L domains and clinically significant changes in the EQ index and EQ VAS based on minimal important change (MIC) thresholds (EQ index > 0.074; EQ VAS > 9.2 for improvements and > 6.5 for deterioration). Values are the number of participants (normal CTA/abnormal CTA). Three-month changes are relative to baseline (n = 91); 12-month changes are relative to the three-month assessment. Both three- and twelve-month data were available for a subset of the cohort (n = 52).

EQ-5D-3L measure	Worsened at 3 mo	Improved at 3 mo	Worsened at 12 mo	Improved at 12 mo
Any EQ-5D-3L domain	15 (14/1)	19 (19/0)	21 (19/2)	17 (16/1)
Anxiety/depression	8 (7/1)	5 (5/0)	9 (8/1)	7 (6/1)
Usual activities	3 (3/0)	4 (4/0)	8 (8/0)	3 (3/0)
Pain/discomfort	3 (3/0)	7 (7/0)	8 (6/2)	6 (6/0)
Mobility	2 (2/0)	6 (6/0)	8 (6/2)	4 (4/0)
Self-care	3 (3/0)	2 (2/0)	1 (1/0)	2 (2/0)
**Clinically significant change (MIC)**				
EQ index	11 (10/1)	9 (9/0)	11 (9/2)	11 (10/1)
EQ VAS	14 (11/3)	25 (24/1)	15 (14/1)	9 (7/2)

#### 3.3.2 Changes at 12 months

Comparing the three-month and 12-month HRQoL, 21 (40%) out of 52 participants reported deterioration and 17 (33%) reported improvements in one or more EQ-5D-3L domains. MIC-significant change was approximately balanced for the EQ index (11 worsened, 11 improved) but weighted toward worsening for the EQ VAS (15 worsened, 9 improved). Full counts are presented in Table 3.

### 3.4 Outcomes of surgically treated patients

Two of the patients with abnormal CTA findings were surgically treated, both experiencing worsening of their HRQoL at three months compared to baseline. One of these patients had a small, 2 mm posterior communicating artery aneurysm, which was ultimately operated on following comprehensive discussion of all treatment options due to the psychological distress of the patient. Following screening but prior to surgical treatment, the patient reported worsening in 15D domains of mobility, usual activities, discomfort and symptoms, depression, sexual function. The patient had a history of psychiatric illness and needed further psychiatric rehabilitation following the surgery. The other surgically treated patient had a 7 mm middle cerebral artery aneurysm that was operated on. There were no complications and the patient was discharged on the second postoperative day. Three months after the screening results but before surgery, the patient reported increased disability in domains of excretion, mental functioning and vitality. At a 5-year follow-up questionnaire, excretion and mental functioning had returned to baseline.

### 3.5 Linear mixed model

For the EQ index, no statistically significant effects were found for timepoint (baseline vs. three months; p = 0.737) or CTA result (normal vs. abnormal; p = 0.231). No interaction between Timepoint x CTA was observed (p = 0.582), indicating that the HRQoL trajectory for those with abnormal or normal findings on the CTA did not differ significantly. Likewise, no significant effects for the timepoint (p = 0.630) or CTA result (p = 0.651) were observed for the EQ VAS, and no Timepoint x CTA result interaction (p = 0.077) was found.

## 4 Discussion

A recognized concern of the implementation of UIA screening is that it could unnecessarily expose participants to the psychological burden of both participating in the screening process and the knowledge of harboring an aneurysm. In our cohort, a substantial proportion of participants experienced deterioration in HRQoL domains related to mental health, regardless of whether the screening revealed an abnormality. Despite this, the overall impact of screening on aggregate HRQoL metrics (EQ-index and EQ VAS) did not reach significance.

Our finding is consistent with a recent prospective study of 461 individuals screened for familial UIAs. The study reported no significant negative effect on HRQoL as assessed by the EQ-5D index and EQ VAS over one year (Mensing et al., 2023). EQ-5D scores improved modestly at 12 months, anxiety remained stable, and depression increased slightly but stayed below general population levels. Only one of 129 participants who answered the question one year after screening expressed regret. Another study from Norway assessed HRQoL in participants of the population-based Tromsø Study, using questionnaires completed as part of the health survey before participants were invited to MRA screening as a baseline (Rosenlund et al., 2024). At follow-up several years later, EQ VAS was slightly lower among participants with UIAs than among screen-negative controls, while other HRQoL parameters were unaffected. Due to the long follow-up (median 32-55 months), however, the immediate transient effects of screening were not assessed.

Upon closer examination of participant-level changes between baseline and three-month follow-up scores, more than half of participants in our study experienced deterioration in one or more 15D domains. Of the affected domains, worse scores were seen particularly in depression, sleep and anxiety. A similar pattern was evident in the EQ-5D-3L responses, including worsening in the anxiety/depression dimension in eight participants. These shifts occurred in comparable proportions among those with normal and abnormal CTA findings, suggesting that the screening process itself may contribute to transient psychological distress. This phenomenon, often termed “scanxiety,” has been well documented in cancer screening and surveillance programs (Derry-Vick et al., 2023, 2025; Bui et al., 2021) and has also been observed in patients undergoing follow-up imaging for known cerebrovascular lesions (Kamphuis et al., 2024).

The presence of scanxiety may also partially explain the improvement of HRQoL in a notable portion of patients with normal CTA findings. Over half of the cohort reported gains in one or more 15D domains at three months, predominantly among those with negative findings. While improvements in mental health-related domains (anxiety, depression, sleep) may be linked to reassurance following a normal scan, gains in unrelated domains, such as symptoms, discomfort, vitality, and breathing, are more difficult to attribute directly to screening. These seemingly nonspecific fluctuations raise questions about the sensitivity and specificity of traditional multidimensional HRQoL instruments in capturing the subtle psychosocial effects of screening programs.

Our study has limitations. The small number of participants with abnormal findings limited statistical power for subgroup analyses. Additionally, the small sample size and lack of a pre-invitation baseline makes the study exposed to the effect of an interpretive bias. HRQoL data at 12 months were available for only a subset of the cohort. However, the 12-month questionnaire was sent only to the 2023–2024 wave, of whom 88% responded, and responders did not differ from non-responders. Most importantly, the 15D and EQ-5D-3L instruments incorporate numerous domains beyond mental health, which may dilute detection of any specific psychological impact of screening. The observed pattern in which participants HRQoL domains move in opposite directions with little net change in summary scores highlights a broader challenge: traditional HRQoL questionnaires may be increasingly influenced by a wide array of everyday life circumstances unrelated to the medical intervention under study. This can make it difficult to isolate the true effect of screening on well-being. Lastly, the results of the study apply to self-selected 50- to 60-year-old Finnish female smokers. As such, they may not be generalizable to, for example, men or to other nationalities.

## 5 Conclusion

In this cohort of female smokers undergoing UIA screening, aggregate EQ-5D measures showed no significant overall impairment in health-related quality of life. However, individual-level analyses revealed heterogeneous changes, including worsening of anxiety and depression in a notable proportion of participants, irrespective of screening outcome. Traditional HRQoL instruments, while valuable, may not be optimally suited to detecting the specific psychosocial consequences of screening, as they are sensitive to numerous factors unrelated to screening. Additionally, strong conclusions cannot be drawn with regards to HRQoL due to the small sample size. Future studies would benefit from pre-invitation baseline assessments of HRQoL and from incorporating targeted anxiety measures (such as GAD-7 or HADS) alongside standard HRQoL tools to better characterize these effects.

## Declaration of competing interest

The authors declare that they have no known competing financial interests or personal relationships that could have appeared to influence the work reported in this paper.
