# Supplementary material for: Individual-level quality-of-life changes are common after aneurysm screening in women: A prospective cohort study
Source: Brain Spine. 2026 Oct 23;6:107491. doi: 10.1016/j.bas.2026.107491 (PMC13720993; doi:10.1016/j.bas.2026.107491)
Supplement: Supplementary file 1 [file mmc1.docx]

Supplementary Table S1A. Baseline characteristics of participants who did and did not return the 3-month questionnaire. Values are median (IQR) or n (%).

| **Variable** | **Responders (n = 92)** | **Non-responders (n = 16)** | **P-value** |
| --- | --- | --- | --- |
| Age at CTA, y | 57.1 (6.1) [n=92] | 53.3 (5.2) [n=16] | 0.003* |
| BMI, kg/m² | 27.3 (8.6) [n=89] | 26.8 (8.0) [n=16] | 0.834 |
| Cigarettes per day | 15.0 (7.0) [n=91] | 11.0 (5.0) [n=16] | 0.623 |
| Pack-years | 22.5 (18.8) [n=90] | 18.0 (13.9) [n=14] | 0.269 |
| Fagerström score | 3 (2) [n=87] | 2 (2) [n=16] | 0.655 |
| Education level (1–6) | 3 (2) [n=92] | 4 (2) [n=16] | 0.803 |
| Self-rated health (1–5, higher = worse) | 3 (1) [n=92] | 2 (1) [n=16] | 0.667 |
| Baseline EQ index | 0.73 (0.27) [n=89] | 0.73 (0.29) [n=16] | 0.680 |
| Baseline EQ VAS | 76.0 (20.0) [n=91] | 81.0 (20.0) [n=16] | 0.175 |
| Hypertension | 47 (51.1%) | 8 (50.0%) | 0.936 |
| Hypercholesterolaemia | 33 (35.9%) | 6 (37.5%) | 0.900 |
| Asthma and/or COPD | 19 (20.7%) | 5 (31.2%) | 0.343 |
| Depression/psychiatric illness | 16 (17.4%) | 4 (25.0%) | 0.491 |
| Cancer | 8 (8.7%) | 2 (12.5%) | 0.641 |
| Abnormal CTA | 10 (10.9%) | 3 (18.8%) | 0.405 |

*P < 0.05

Supplementary Table S1B. 12-month questionnaire responders vs non-responders. Values are median (IQR) or n (%).

| **Variable** | **Responders (n = 57)** | **Non-responders (n = 8)** | **P-value** |
| --- | --- | --- | --- |
| Age at CTA, y | 56.1 (5.2) [n=57] | 56.5 (4.0) [n=8] | 0.889 |
| BMI, kg/m² | 28.7 (10.2) [n=55] | 29.1 (11.8) [n=8] | 0.812 |
| Cigarettes per day | 12.5 (5.0) [n=57] | 15.0 (6.9) [n=8] | 0.663 |
| Pack-years | 20.0 (17.2) [n=57] | 21.9 (18.3) [n=8] | 0.772 |
| Fagerström score | 2 (2) [n=55] | 3 (2) [n=8] | 0.975 |
| Education level (1–6) | 2 (2) [n=57] | 3 (2) [n=8] | 0.934 |
| Self-rated health (1–5, higher = worse) | 3 (1) [n=57] | 3 (0) [n=8] | 0.983 |
| Baseline EQ index | 0.67 (0.21) [n=56] | 0.60 (0.22) [n=8] | 0.211 |
| Baseline EQ VAS | 70.0 (20.0) [n=57] | 70.0 (20.0) [n=8] | 0.421 |
| 3-month EQ index | 0.67 (0.20) [n=52] | 0.60 (0.24) [n=6] | 0.455 |
| 3-month EQ VAS | 76.0 (15.0) [n=52] | 65.0 (27.5) [n=6] | 0.181 |
| Change in EQ index, 0–3 mo | 0.00 (0.04) [n=51] | 0.00 (0.00) [n=6] | 0.660 |
| Change in EQ VAS, 0–3 mo | 5.0 (10.0) [n=52] | 2.5 (11.2) [n=6] | 0.365 |
| Hypertension | 36 (63.2%) | 4 (50.0%) | 0.700 |
| Hypercholesterolaemia | 20 (35.1%) | 3 (37.5%) | 1.000 |
| Asthma and/or COPD | 13 (22.8%) | 3 (37.5%) | 0.395 |
| Depression/psychiatric illness | 12 (21.1%) | 2 (25.0%) | 1.000 |
| Cancer | 7 (12.3%) | 1 (12.5%) | 1.000 |
| Abnormal CTA | 5 (8.8%) | 1 (12.5%) | 0.561 |
